# Preclinical evaluation of the antiproliferative potential of STI571 in Hodgkin's disease

**DOI:** 10.1038/sj.bjc.6600243

**Published:** 2002-04-22

**Authors:** D Re, C Wickenhauser, T Ahmadi, E Buchdunger, M Kochanek, V Diehl, J Wolf

**Affiliations:** Department of Internal Medicine I, University of Cologne, Joseph-Stelzmann-Str. 9, 50931 Cologne, Germany; Institute of Pathology, University of Cologne, Jospeh-Stelzmann-Str. 9, 50931 Cologne, Germany; Novartis Pharma AG, Oncology Research, 4002 Basel, Switzerland

**Keywords:** Hodgkin and Reed Sternberg cells, STI571, c-*kit* (CD117)

## Abstract

STI571 is a selective tyrosine kinase inhibitor with proven therapeutic potential in malignancies expressing c-*kit*. A strong c-*kit* and stem cell factor expression was detected in the Hodgkin and Reed Sternberg cell line L1236, but not in 20 primary cases of classical Hodgkin's disease. Proliferation of L1236 cells was neither affected by addition of stem cell factor nor by neutralising anti-stem cell factor antibodies or STI571. Results suggest that patients with Hodgkin's disease may not benefit from therapy with STI571.

*British Journal of Cancer* (2002) **86**, 1333–1335. DOI: 10.1038/sj/bjc/6600243 www.bjcancer.com

© 2002 Cancer Research UK

## 

The malignant cells in classical Hodgkin's disease (cHD), the Hodgkin and Reed Sternberg cells (H-RS), are mostly derived from germinal centre B-cells as indicated by somatic mutations within their rearranged immunoglobulin genes (Küppers and Rajewsky, 1998). Nevertheless, H-RS cells phenotypically exert several characteristics not typical for B-cells. They lack expression of most B-cell specific proteins (Drexler, 1992) and, instead, express proteins restricted to other lineages or to immature precursor cells (Cossman *et al*, 1998). Expression of c-*kit* as a marker of an immature phenotype, for instance, has been described in H-RS cells in cHD previously (Pinto *et al*, 1994).

The tyrosine kinase inhibitor STI571 has been shown to selectively inhibit the receptor tyrosine kinases v-*abl* and bcr–abl, the platelet derived growth factor receptor (PDGFR) and the receptor for the stem cell factor, i.e. c-*kit* (Druker *et al*, 1996; Buchdunger *et al*, 2000; Heinrich *et al*, 2000). This specific activity of STI571 is already exploited for tumour control in chronic myelogeneous leukaemia (Druker *et al*, 2001) and soft tissue sarcomas (Joensuu *et al*, 2001). It recently has been proposed (Buchdunger *et al*, 2000) that STI571 may also be effective in other malignancies expressing receptors for PDGF or c-*kit*. We here evaluate the expression of c-*kit* in HD derived cell lines and cases of cHD in order to test the therapeutic potential of STI571 for cHD.

## MATERIALS AND METHODS

### Cell lines and pathological specimen

The characteristics of the six HD derived cell lines are summarised by Drexler (1993) and Wolf *et al* (1996). L1309 is an EBV immortalised lymphoblastoid cell line from a healthy donor. M07e is a myeloid leukaemia cell line purchased at DSMZ (Braunschweig, Germany). Twenty primary cases of classical HD (seven mixed cellularity, nine nodular sclerosis, two lymphocyte depleted, two lymphocyte rich classical) and a case of gastrointestinal mesenchymal stromal tumour were analysed by immunohistochemistry. Immunohistochemistry was performed on paraffin embedded formalin-fixed specimen according to standard protocols using a polyclonal rabbit anti-human c-*kit* antibody (DAKO, Hamburg, Germany).

### Flow cytometry

Hodgkin's disease derived cell lines were incubated with a phycoerythrin conjugated mouse anti-human CD117 monoclonal antibody (clone 95C3; AnDerGrub, Austria) following the instructions of the manufacturer and analysed on a Becton-Dickinson FACS Calibur.

### Proliferation assay

Hodgkin's disease derived cell lines and LCL1309 were plated in 96-well flat bottom culture plates at a density of 20 000 cells per well. M07e cells were plated at 50 000 cells per well and cultured in the presence of stem cell factor (SCF) (200 ng ml^−1^; R&D, Germany). Recombinant human SCF, neutralising anti-human SCF antibody (dissolved in phosphate buffered saline; R&D) or STI571 (dissolved in DMSO; provided by Dr Elizabeth Buchdunger, Novartis Pharma, Basel, Switzerland) was added according to the respective experimental setup. At 48 h MTT was added to each well and cells were lysed after 2 h following the instructions of the manufacturer (TACS assay; R&D).

### Polymerase chain reaction and sequence analysis

High molecular weight DNA was extracted from L1236 cells according to standard protocols. Amplification and sequencing of exon 11 and 17 was performed at 55°C as described previously (Re *et al*, 2001) using published oligonucleotides (Tian *et al*, 1999).

### Human SCF immunoassay

Amounts of SCF were quantified in 24 h supernatants from cell lines using a commercially available ELISA kit (Quantakine; R&D).

## RESULTS AND DISCUSSION

### Analysis of c-*kit* and SCF expression in HD derived cell lines

Using FACS analysis, a strong cell surface expression of c-*kit* was detected in the HD derived cell line L1236 while c-*kit* expression was absent in five other HD derived cell lines (L428, KM-H2, L591, Hdlm-2, L540) (Table 1

**Table 1:**
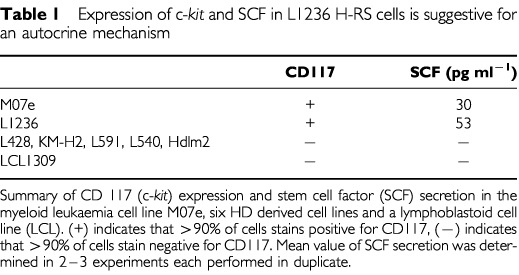
Expression of c-*kit* and SCF in L1236 H-RS cells is suggestive for an autocrine mechanism

). Secretion of the c-*kit* ligand SCF was tested in the supernatant of all six HD derived cell lines and controls. Low amounts of SCF were detected in L1236 and M07e cell cultures (53 and 30 pg ml^−1^, respectively). Detection of both SCF and c-*kit* in L1236 cells suggested an autocrine mechanism of growth control in L1236 cells.

### Treatment of HD-derived cell lines with SCF, anti-SCF antibodies and STI571

**Figure 1:**
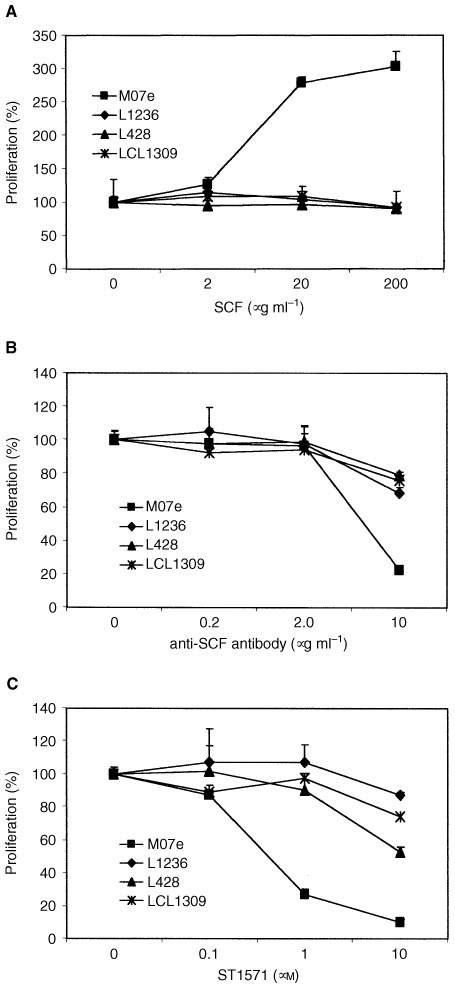
Proliferation of c-*kit* positive L1236 cells is not influenced upon stimulation with SCF, anti-SCF antibodies or the c-*kit* inhibitor STI571. Forty-eight-hours MTT proliferation assay with the HD derived cell lines L1236 (c-*kit*^+^/SCF^+^) and L428 (c-*kit*^−^/SCF^−^), the lymphoblastoid cell line 1309 (c-*kit*^−^/SCF^−^) as negative control and the myeloid cell line M07e (c-*kit*^+^/SCF^+^) as postivie control. Incubation of cell lines with increasing doses of (**A**) stem cell factor (SCF); (**B**) neutralising anti-SCF antibody; (**C**) tyrosine kinase inhibitor STI571. Each experiment was performed in triplicate and repeated at least three times. Mean value of proliferation and single standard deviation of representative experiments are shown.

In order to further characterise the postulated autocrine role of the c-*kit*/SCF interaction for proliferation and viability of L1236 cells, a MTT (dimethylthiazol–diphenyltetrazolium bromide) based assay was performed with L1236 cells and controls (M07e was used for positive control and cell lines L428 and LCL1309 for negative control). As shown in Figure 1A

, addition of SCF to cell cultures stimulated growth of control cells M07e but not of L1236 cells. Accordingly, after addition of a neutralising anti-SCF antibody to the cell cultures, proliferation of M07e but not of L1236 cells was inhibited (Figure 1B).

Mutant constitutively active c-*kit* receptors that are found e.g. in mast cell disease (Longley *et al*, 1996) may cause an SCF independent proliferation of c-*kit* expressing cells. Since these activating c-*kit* mutations mostly have been detected in exon 11 and exon 17, we performed L1236 DNA sequence analysis of c-*kit* exon 11 and 17 (GenBank accession number 1817732: base pair 75662–75788 and 81257–81463). These experiments revealed germ line configuration of both exons (data not shown). It is thus concluded, that SCF independence of L1236 proliferation is unlikely to be due to DNA mutations of the c-*kit* gene.

When cells were treated with the tyrosine kinase inhibitor STI571, positive controls but not H-RS cells showed a marked decrease of c-*kit* dependent proliferation at STI571 doses of 0.1 to 1 μmol l^−1^ (Figure 1C). With increasing doses of STI571, L1236 cells as well as negative controls L428 and LCL1309 showed reduction of proliferation rate possibly due to unspecific toxic effects.

### Immunostaining of H-RS cells* in situ* for c-*kit* expression

Twenty primary cases of cHD were immunostained using a monoclonal antibody specific for c-*kit*. A gastrointestinal mesenchymal stromal tumour with known strong cell surface expression of c-*kit* (Hirota *et al*, 1998) was used as positive control. Absence of c-*kit* expression was found in H-RS cells of all twenty cases. This negative result was somewhat surprising as Pinto *et al* (1994) reported a partially strong c-*kit* expression in most H-RS cells of 11 out of 17 cases of cHD. The c-*kit* receptor protein expression was selectively detected in cases of cHD and anaplastic large cell lymphoma while it was absent in cases of non-Hodgkin's lymphoma. It therefore has been speculated, that c-*kit* may be activated in an autocrine or paracrine fashion and regulate growth of c-*kit* expressing neoplastic cells. In contrast, our results suggest, that H-RS cells commonly do not express c-*kit*. These immunohistochemical analyses are well conceivable with our *in vitro* experiments suggesting, that activation of c-*kit* is not even involved in control of proliferation of c-*kit* positive H-RS cells of the cell line L1236. It is therefore concluded, that administration of the c-*kit* inhibitor STI571 may not be beneficial for patients with cHD.
